# A rare *Waxy* allele coordinately improves rice eating and cooking quality and grain transparency

**DOI:** 10.1111/jipb.13010

**Published:** 2020-12-29

**Authors:** Changquan Zhang, Yong Yang, Shengjie Chen, Xueju Liu, Jihui Zhu, Lihui Zhou, Yan Lu, Qianfeng Li, Xiaolei Fan, Shuzhu Tang, Minghong Gu, Qiaoquan Liu

**Affiliations:** ^1^ Key Laboratory of Crop Genomics and Molecular Breeding of Jiangsu Province, State Key Laboratory of Hybrid Rice Yangzhou University Yangzhou 225009 China; ^2^ Key Laboratory of Plant Functional Genomics of the Ministry of Education/Jiangsu Key Laboratory of Crop Genetics and Physiology/Co‐Innovation Center for Modern Production Technology of Grain Crops of Jiangsu Province Yangzhou University Yangzhou 225009 China

**Keywords:** allelic variation, amylose content (AC), eating and cooking quality (ECQ), grain appearance, *Oryza sativa L.*, *Waxy*

## Abstract

In rice (*Oryza sativa*), amylose content (AC) is the major factor that determines eating and cooking quality (ECQ). The diversity in AC is largely attributed to natural allelic variation at the *Waxy* (*Wx*) locus. Here we identified a rare *Wx* allele, *Wx* ^*mw*^, which combines a favorable AC, improved ECQ and grain transparency. Based on a phylogenetic analysis of *Wx* genomic sequences from 370 rice accessions, we speculated that *Wx* ^*mw*^ may have derived from recombination between two important natural *Wx* alleles, *Wx* ^*in*^ and *Wx* ^*b*^. We validated the effects of *Wx* ^*mw*^ on rice grain quality using both transgenic lines and near‐isogenic lines (NILs). When introgressed into the *japonica* Nipponbare (NIP) background, *Wx* ^*mw*^ resulted in a moderate AC that was intermediate between that of NILs carrying the *Wx* ^*b*^ allele and NILs with the *Wx* ^*mp*^ allele. Notably, mature grains of NILs fixed for *Wx* ^*mw*^ had an improved transparent endosperm relative to soft rice. Further, we introduced *Wx* ^*mw*^ into a high‐yielding *japonica* cultivar via molecular marker‐assisted selection: the introgressed lines exhibited clear improvements in ECQ and endosperm transparency. Our results suggest that *Wx* ^*mw*^ is a promising allele to improve grain quality, especially ECQ and grain transparency of high‐yielding *japonica* cultivars, in rice breeding programs.

## Graphical abstract

In the present study, a rare *Wx* allele, *Wx^mw^* which may have derived from recombination between two important natural *Wx* alleles in rice, was identified as a promising allele to improve grain quality, especially cooked rice taste and grain transparency of high‐yielding japonica cultivars, in rice breeding programs.

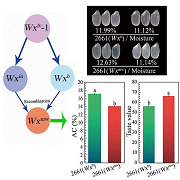

## INTRODUCTION

Rice (*Oryza sativa* L.) is a major staple food and the main source of dietary carbohydrates for half of the world's population. To meet the food requirements for the growing human population, continuous improvements in both rice yield and grain quality will be necessary (Tilman et al., 2011; Custodio et al., 2019). As rice is commonly consumed as cooked intact grains, both the eating and cooking quality (ECQ) and grain transparency (a measure of rice commercial and appearance quality) have been the key traits selected in released cultivars (Zhou et al., 2020).

The main macromolecules accumulating in a polished rice grain are starches (amounting up to 90% by dry weight). The starch composition of the endosperm, indicated by the grain amylose content (AC), is a key factor determining the physicochemical properties and ECQ of rice grains (Fitzgerald et al., 2009; Li et al., 2016). Rice cultivars are routinely classified according to their AC: high (>25%), intermediate (20%–25%), low (10%–19%), very low or soft (3%–9%), and waxy or glutinous (<2%). Generally, cooked rice grains with a low or intermediate AC exhibit an elastic, sticky, and glossy texture, whereas high AC varieties are characterized by firm and separated grains after cooking (Li et al., 2016). Notably, consumer preferences for rice ECQ differ by region and culture. For instance, individuals from China, Japan, and Korea tend to prefer rice with low AC and sticky ECQ, whereas individuals from India and Bangladesh prefer high AC rice, with firm, separate grains when cooked (Misra et al., 2018; Custodio et al., 2019).

In the rice endosperm, amylose synthesis is largely regulated by the *Wx* locus located on chromosome 6, which encodes granule‐bound starch synthase I (GBSSI) (Wang et al., 1990). The diversity of AC is largely attributable to allelic variation at the *Wx* locus (Tian et al., 2009; Biselli et al., 2014; Zhang et al., 2019a). To date, at least eight *Wx* alleles, *Wx* ^*lv*^, *Wx* ^*a*^, *Wx* ^*in*^, *Wx* ^*b*^, *Wx* ^*op/hp*^, *Wx* ^*mq*^, *Wx* ^*mp*^, and *wx*, have been shown to be associated with the five possible amylose types observed in rice cultivars (Cai et al., 1998; Sato et al., 2002; Larkin and Park, 2003; Wanchana et al., 2003; Mikami et al., 2008; Liu et al., 2009; Yang et al., 2013; Zhang et al., 2019a). Most of these alleles have been successfully incorporated into modern rice cultivars, where *Wx* ^*op/hp*^, *Wx* ^*mq*^, and *Wx* ^*mp*^ (controlling low to very low AC) have been introgressed to breed good ECQ varieties known as soft rice. However, grain appearance for these soft rice varieties with AC values commonly lower than <13% is usually dull or opaque, and thus may not meet the commercial or appearance quality desired by consumers (Liu et al., 2009; Li et al., 2018). Therefore, there is a need to screen rice accessions and mine novel *Wx* alleles with appropriate AC (such as 13%–14%) from natural germplasm collections to help generate new cultivars with both good ECQ and grain transparency.

In the present study, we identified and characterized a novel *Wx* allele, *Wx* ^*mw*^, from a low AC rice landrace. We present here a detailed analysis of *Wx* ^*mw*^ with respect to amylose synthesis as well as its evolutionary relationship with other *Wx* alleles. Our results suggest that *Wx* ^*mw*^ will be instrumental in breeding new rice varieties, especially in *japonica* rice, with improved ECQ and a transparent grain appearance.

## RESULTS

### A landrace rice with relatively low AC and good transparency

**Figure 1:**
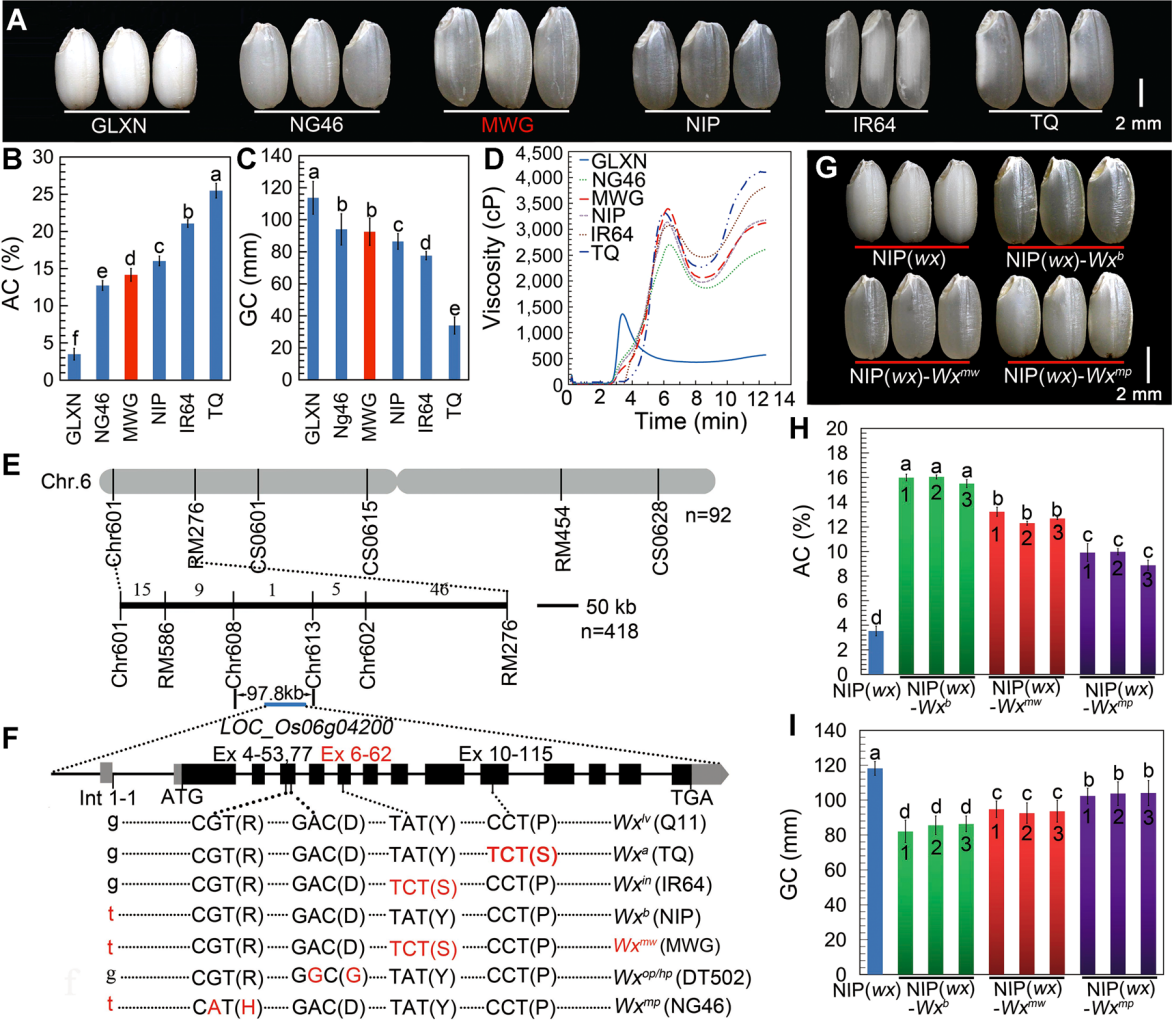
Map‐based cloning and functional characterization of *Wx*^*mw*^ (**A–D**) Comparison of grain appearance (**A**), amylose content (AC) (**B**), gel consistency (GC) (**C**) and rapid viscosity analysis (RVA) profiles (**D**), between Mowanggu (MWG) and other rice varieties carrying the various *Wx* alleles shown in panel (**F**). GLXN (Guanglingxiangnuo, glutenous rice with the null *wx* allele); NG46 (Nangeng46) and NIP (Nipponbare) are *japonica*, while IR64 and TQ (Teqing) are *indica* varieties. (**E**) Linkage analysis and mapping of *Wx* ^*mw*^. (**F**) Genomic structure of *Wx* and alignment of several major polymorphic sequences among different *Wx* alleles. Representative rice cultivars for each *Wx* allele are listed in brackets. (**G–I**) Phenotypes of mature brown rice (**G**), AC (**H**) and GC (**I**) for transgenic rice lines and their wild type progenitor NIP(*wx*). NIP(*wx*)‐*Wx* ^*b*^, NIP(*wx*)‐*Wx* ^*mw*^, and NIP(*wx*)‐*Wx* ^*mp*^ represent transgenic lines with intact *Wx* ^*b*^, *Wx* ^*mw*^, or *Wx* ^*mp*^ transgenes, respectively, in the glutinous NIP(*wx*) background. All transgenic lines were homozygous and from the T_3_ generation. Values labeled with different lowercase letters are significantly different by one‐way analysis of variance (ANOVA) multiple comparison (*P* < 0.05). Error bars indicate *SD*, *n* = 3.

After screening many rice accessions for desirable combinations of grain traits, we discovered that the rice landrace Mowanggu (MWG) from southern China had a relatively low AC (14.1%). Under natural air‐drying conditions, MWG grains exhibited a more transparent endosperm, slightly higher AC but similar soft gel consistency (GC) when compared with the soft *japonica* rice variety Nangeng 46 (NG46) (Figure 1A–C). NG46 is now a famous released cultivar in China that carries the *Wx* ^*mp*^ allele and shows very low AC (12.73%) and good ECQ. With respect to pasting characteristics, MWG rice flours and starches displayed a high breakdown and low setback value, similar to those seen in NG46 (Figures 1D, S1; Table S2), suggestive of a good ECQ. Thus, we hypothesized that MWG may constitute a potentially novel rice type with higher AC, better grain transparency but similar ECQ properties relative to the soft rice NG46.

### Mowanggu harbors a novel *Wx* allele, *Wx* ^*mw*^

To identify the putative gene responsible for low AC in MWG grains, we crossed the MWG variety to the *japonica* cultivar Nipponbare (NIP), which carries the *Wx* ^*b*^ allele and has an AC of 16.02%. We phenotyped 500 individuals from the resulting segregating F_2_ population and selected 92 individuals with MWG‐like low AC for genetic mapping. We determined that the causal locus responsible for the low AC mapped to the short arm of chromosome 6. We then selected 418 individuals with low AC from 2 000 individuals derived from the F_3_ progeny of informative F_2_ lines for fine mapping, which narrowed down the location of the causal locus between markers Chr 608 and Chr 613 (Figure 1E). The *Wx* locus was included in the mapping interval, highlighting *Wx* as an obvious candidate (Figure 1E–F). We therefore sequenced the entire *Wx* gene (8 073 bp) in the MWG background, and identified a single A‐to‐C substitution on exon 6 (EX6**–**62) relative to the *Wx* ^*b*^ allele from NIP (Figure 1F). This A‐to‐C substitution was also present in the *Wx* ^*in*^ allele (from IR64, associated with intermediate AC). However, *Wx* ^*in*^ also included another single nucleotide polymorphism (SNP) in the first intron (Int 1–1) that distinguished *Wx* ^*in*^ (with a G) from *Wx* from MWG (with a T). Furthermore, the *Wx* allele from MWG did not share any functional SNPs with the other sequenced *Wx* ^*lv*^, *Wx* ^*a*^, *Wx* ^*op*^, or *Wx* ^*mp*^ alleles (Figure 1F).

These results indicated that the newly cloned *Wx* allele from the MWG accession combines the T polymorphism at the Int 1‐1 position, as in *Wx* ^*b*^, with the C polymorphism in Ex 6‐62, as in *Wx* ^*in*^. Therefore, the relatively low AC exhibited by MWG may be attributable to the combination of these two SNPs. We will refer to the *Wx* allele from MWG as the novel haplotype *Wx* ^*mw*^ (*Wx* from Mowanggu). Previous association analyses of diverse rice collections had demonstrated a significant association between low AC and both the Int 1–1 and Ex 6–62 SNPs at the *Wx* locus (Larkin and Park, 2003; Hoai et al., 2014). However, how the *Wx* ^*mw*^ haplotype affects rice grain quality has not been investigated. In the following experiments, we thus compared the functional consequences associated with the *Wx* ^*mw*^ allele with those of the *Wx* ^*b*^ and *Wx* ^*mp*^ alleles, also resulting in low AC.

### *Wx* ^*mw*^ contributes to low AC and improved rice ECQ

To validate the role of the novel *Wx* ^*mw*^ allele in establishing a low AC content, we introduced three constructs (Figure S2), carrying the entire *Wx* ^*mw*^, *Wx* ^*b*^, and *Wx* ^*mp*^ genomic regions from MWG, NIP and NG46, respectively, into the glutinous near‐isogenic line (NIL) NIP(*wx*), carrying the null *wx* allele in the NIP background. Under natural air‐drying conditions, transgenic grains carrying the *Wx* ^*mw*^ transgene showed a better transparency than transgenic grains with the *Wx* ^*mp*^ transgene (Figure 1G). Transgenic NIP(*wx*)‐*Wx* ^*mw*^ grains had a significantly lower AC (12%–13%) relative to NIP(*wx*)‐*Wx* ^*b*^ transformants, although their AC remained higher than that of NIP(*wx*)‐*Wx* ^*mp*^ transgenic grains (Figure 1H). The gradient texture of rice grains followed a negative correlation with AC values across the transformants, as evidenced by the GC data (Figure 1H, I), but we observed no significant differences in gelatinization temperature (GT) between NIP(*wx*)‐*Wx* ^*mw*^ and NIP(*wx*)‐*Wx* ^*mp*^ transgenic grains (Table S3).

**Figure 2:**
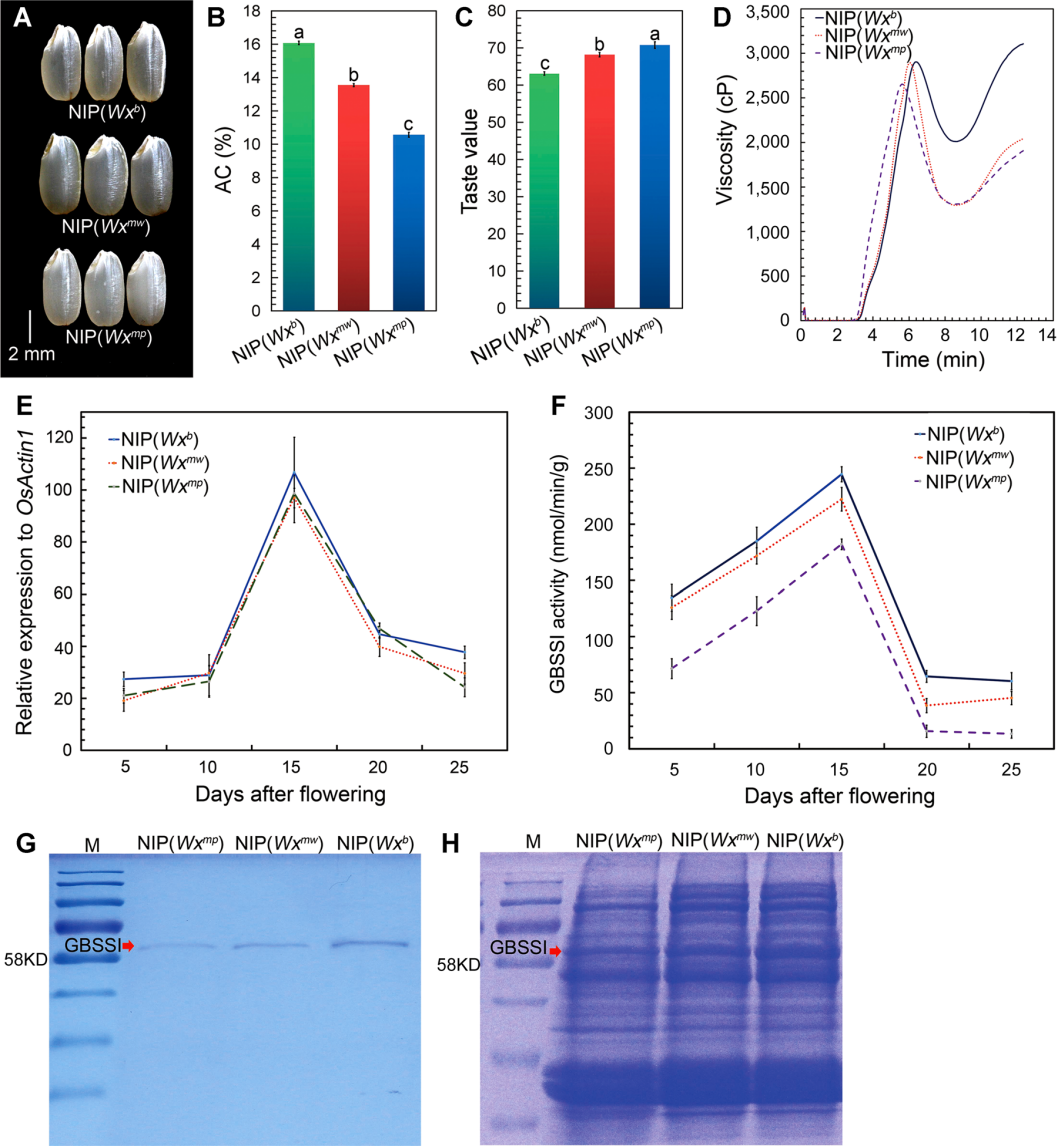
Comparison of grain qualities and *Wx* expression among near‐isogenic lines (NILs) (**A**) Appearance of brown rice. (**B**) Amylose content (AC) of rice flours. (**C**) Taste value of cooked rice. (**D**) Rapid viscosity analysis (RVA) profiles of rice flours. (**E–F**) Relative expression levels of *Wx* messenger RNA (**E**) and granule‐bound starch synthase I (GBSSI) activity (**F**) in developing seeds at various days after flowering. (**G–H**) Sodium dodecyl sulfate‐polyacrylamide gel electrophoresis (SDS‐PAGE) assays of starch granule‐bound GBSSI (**G**) and total seed proteins (**H**) from immature seeds 15 d after flowering. NIP(*Wx* ^*mw*^) and NIP(*Wx* ^*mp*^) are the near‐isogenic lines introgressed with the *Wx* ^*mw*^ or *Wx* ^*mp*^ alleles, respectively, in the *japonica* Nipponbare (NIP(*Wx* ^*b*^)) background. Values labeled with different lowercase letters are significantly different by one‐way analysis of variance (ANOVA) multiple comparison (*P* < 0.05). Error bars indicate *SD*, *n* = 3.

In a complementary approach, we generated two NILs, NIP(*Wx* ^*mw*^) and NIP(*Wx* ^*mp*^), in the NIP background, by introgressing the *Wx* ^*mw*^ and *Wx* ^*mp*^ alleles from MWG and NG46, respectively. We confirmed that both NILs carried only a small segment around the *Wx* region from the donor genomes with published molecular markers (Zhang et al., 2011), and exhibited an overall plant morphology that was comparable with that of their recurrent parent NIP(*Wx* ^*b*^) (Figure S3). As expected, brown rice from the NIP(*Wx* ^*mw*^) line exhibited a slightly darker endosperm when compared with NIP(*Wx* ^*b*^) grains, but had improved appearance relative to NIP(*Wx* ^*mp*^) grains (Figure 2A). In addition, NIP(*Wx* ^*mw*^) grains had an AC that was intermediate between NIP(*Wx* ^*b*^) and NIP(*Wx* ^*mp*^) (Figure 2B), and exhibited very similar soft GC values and rapid viscosity analysis (RVA) curves to NIP(*Wx* ^*mp*^) grains (Figure 2D; Tables S4, S5). Differential scanning calorimetry (DSC) analysis indicated that both NIP(*Wx* ^*mw*^) and NIP(*Wx* ^*mp*^) had lower GT than NIP(*Wx* ^*b*^) (Table S4). Importantly, the sensory property test showed that cooked NIP(*Wx* ^*mw*^) rice grains had a slightly lower taste value relative to cooked NIP(*Wx* ^*mp*^) grains, but ranked higher than cooked NIP(*Wx* ^*b*^) grains (Figure 2C). Together, these results confirmed that *Wx* ^*mw*^ is a novel *Wx* allele with a behavior distinct from the *Wx* ^*b*^ and *Wx* ^*mp*^ alleles, and suggest that *Wx* ^*mw*^ may constitute a suitable genetic material for breeding new rice cultivars with improved grain appearance and ECQ.

### *Wx* ^*mw*^ endosperm has lower GBSSI activity than *Wx* ^*b*^ endosperm

We next compared the expression levels of the *Wx* ^*mw*^, *Wx* ^*b*^, and *Wx* ^*mp*^ alleles in developing seeds from NIP and the respective NILs. All genotypes accumulated similar transcript levels of mature *Wx* messenger RNA (mRNA) during the grain‐filling process (Figure 2E), ruling out *Wx* transcriptional output as the source for the distinct grain properties observed in each genotype. We then turned to the enzymatic activity of GBSSI, the *Wx*‐encoded enzyme. GBSSI activity broadly followed the same pattern over the course of seed development in all three genotypes, but with notable differences as well. Indeed, GSSBI activity derived from the *Wx* ^*mp*^ allele was the lowest measured in our experiments; the *Wx* ^*mw*^ allele in NIP(*Wx* ^*mw*^) resulted in higher GBSSI activity relative to NIP(*Wx* ^*mp*^), but remained lower than that of GBSSI from *Wx* ^*b*^ in the NIP background (Figure 2F).

To determine if GBSSI activity corresponded with protein levels, we performed odium dodecyl sulfate‐polyacrylamide gel electrophoresis analysis of GBSSI (Figure 2G) and total seed proteins (Figure 2H) from immature seeds. GBSSI protein levels clearly varied between NIP and the two NILs, with the most protein accumulating in NIP, followed by NIP(*Wx* ^*mw*^), and finally NIP(*Wx* ^*mp*^) (Figure 2G, H). Collectively, these results indicate that the A–C SNP detected in exon 6 (Ex 6–62) in *Wx* ^*mw*^, which results in a Ser to Tyr substitution (Figure 1E), may decrease GBSSI levels and/or GBSSI activity, leading to a reduction in AC of *Wx* ^*mw*^ endosperm.

### *Wx* ^*mw*^ rice grains are transparent

**Figure 3:**
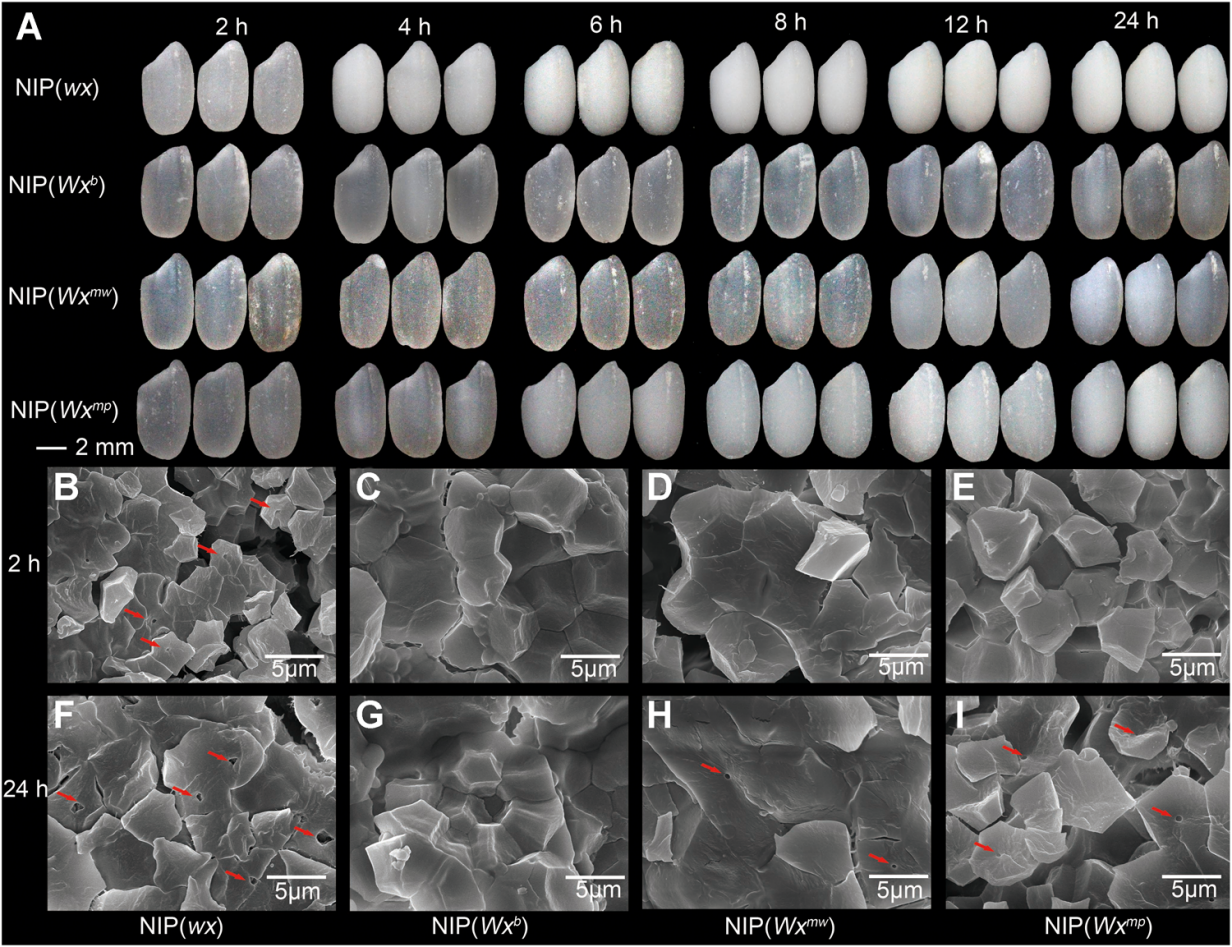
Comparison of endosperm appearance and morphology of grain transverse sections between near‐isogenic lines (NILs) carrying different *Wx* alleles (**A**) Milled rice after incubation at 40 °C for 2, 4, 6, 8, 12, and 24 h. The corresponding moisture contents are listed in Table S7. (**B–E**) Transverse sections of mature grains after 2 h of drying. (**F–I**) Transverse sections of grains after 24 h of drying. Red arrows indicate holes within starch granules. NIP(*wx*), NIP(*Wx* ^*mw*^) and NIP(*Wx* ^*mp*^) are NILs introgressed with the *wx*, *Wx* ^*mw*^ and *Wx* ^*mp*^ alleles, respectively, in the *japonica* Nipponbare (NIP(*Wx* ^*b*^)) background.

The degree of transparency exhibited by milled rice is generally a reflection of AC and moisture content (Zhang et al., 2017; Li et al., 2018). In addition, safe storage of mature rice grains typically requires a moisture content lower than 14%. Therefore, we compared grain appearance along a moisture gradient, generated via gradual air‐drying. Our starting materials consisted of rice grains from three NILs in the NIP background, and carrying the alleles *wx*, *Wx* ^*mp*^ or *Wx* ^*mw*^, respectively. As shown in Figure 3A and Table S7, grains with a high moisture content (over 18%), even glutinous grains from NIP(*wx*), exhibited a transparent appearance. However, glutinous NIP(*wx*) endosperms rapidly became dull or waxy with decreasing seed moisture, while NIP(*Wx* ^*mp*^) grains turned opaque when their moisture content fell below 14%. Notably, NIP(*Wx* ^*mw*^) grains only became opaque when their moisture content dropped below 12%.

We then turned to scanning electron microscope (SEM) observations of transverse grain sections. In either high or extremely low moisture content (2 h and 24 h after drying treatment), we noticed many small holes in the core of starch granules within NIP(*wx*) glutinous endosperm (Figure 3B, F), although we failed to see similar structures in starch granules from transparent NIP(*Wx* ^*b*^) grains (Figure 3C, G). We detected no holes under high moisture conditions in NIP(*Wx* ^*mw*^) or NIP(*Wx* ^*mp*^) grains (Figure 3D, E), although holes did appear later under low moisture. We quantified the area covered by holes in both genotypes: the gaps in NIP(*Wx* ^*mw*^) grain starch granules comprised a smaller area (0.11 ± 0.05 μm^2^) when compared to the gaps in NIP(*Wx* ^*mp*^) rice grains (0.25 ± 0.04 μm^2^) (Figure 3H, I). We observed a similar phenomenon in transgenic rice lines in the NIP(*wx*) background expressing the various *Wx* alleles under investigation here, lending support to the contribution of *Wx* alleles to grain appearance (Figure S4). In agreement with our earlier studies (Zhang et al., 2017, 2019b), these results indicate that the improvement of endosperm transparency in *Wx* ^*mw*^ grains may be due to smaller air pockets in the center of starch granules caused by their slightly higher AC, when compared to soft *Wx* ^*mp*^ rice.

### *Wx* ^*mw*^ may have derived from natural recombination between *Wx* ^*b*^ and *Wx* ^*in*^

**Figure 4:**
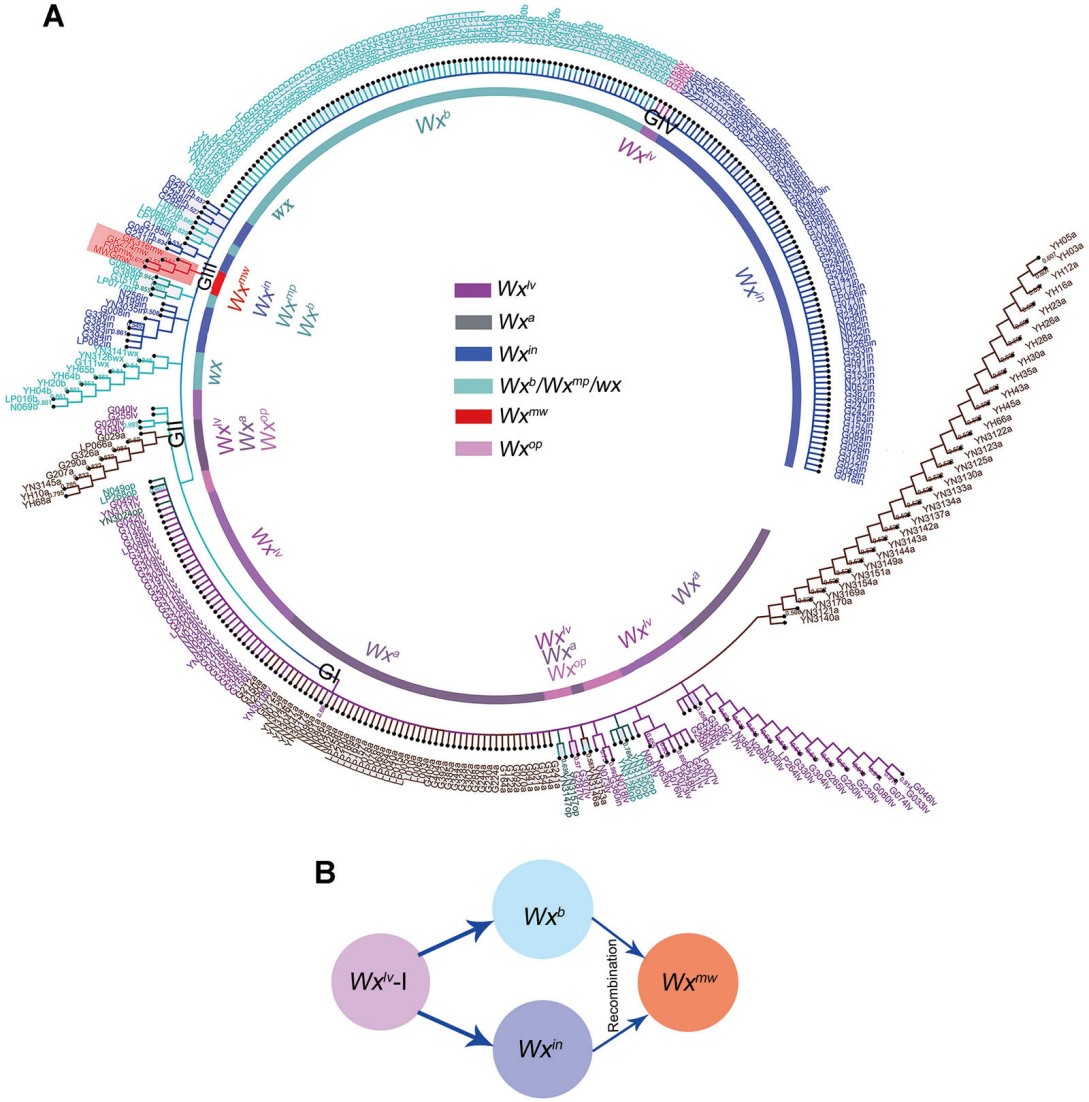
Phylogenetic analysis and proposed evolutionary relationship among various *Wx* alleles in rice (**A**) Neighbor‐joining phylogenetic tree based on full‐length *Wx* genomic sequences from 370 rice accessions. The rice accessions and their *Wx* genotypes are given in Table S8. (**B**) *Wx* ^*mw*^ is derived from the recombination between *Wx* ^*in*^ and *Wx* ^*b*^ alleles. Both alleles are evolved from the type I haplotype (*Wx* ^*lv*^–I) of the ancestral *Wx* ^*lv*^ allele.

To examine the relationship between *Wx* ^*mw*^ and other cloned *Wx* alleles, we compared the full‐length *Wx* gene in a panel of 370 worldwide rice accessions (Zhang et al., 2019a; Table S8). Only four rice accessions (including MWG) belonging to the tropical *japonica* subpopulation carried the *Wx* ^*mw*^ allele. We next constructed a phylogenetic tree of all sequenced *Wx* alleles, which divided the 370 rice accessions into four groups (Figure 4A). Notably, the *Wx* ^*mw*^ allele clustered with the *Wx* ^*in*^ and *Wx* ^*b*^ alleles, indicating a close genetic relationship between these three alleles. We then calculated the average genetic distances between distinct *Wx* alleles. As expected, *Wx* ^*mw*^ appeared to be much closer to both *Wx* ^*in*^ (0.000 52) and *Wx* ^*b*^ (0.000 44) relative to all other alleles (Table S6). In a recent study, we had proposed that the *Wx* ^*b*^ and *Wx* ^*in*^ alleles may have evolved from the same haplotype (*Wx* ^*lv*^–I) from the ancestral *Wx* ^*lv*^ allele (Zhang et al., 2019a). Therefore, we hypothesize here that the *Wx* ^*mw*^ allele may have arisen from a spontaneous recombination between the *Wx* ^*in*^ and *Wx* ^*b*^ alleles during the later stages of rice domestication (Figure 1B). In fact, several studies have determined that the *Wx* locus is a recombination hotspot in rice cultivars (Inukai et al., 2000; Muto et al., 2016).

### Introgression of *Wx* ^*mw*^ into high‐yield cultivars for improved quality

**Figure 5:**
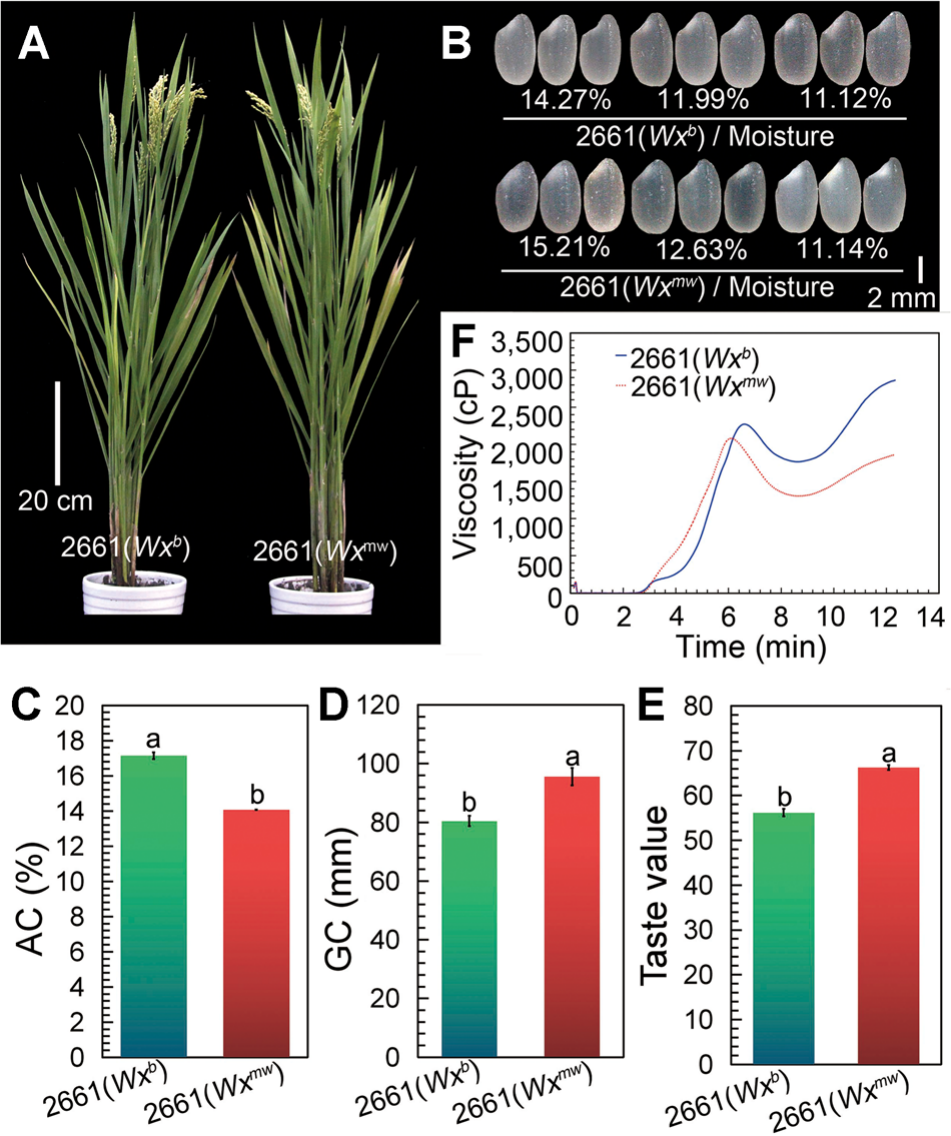
Comparison of plant morphology and grain qualities between 2661(*Wx*^*b*^) and its near‐isogenic line 2661(*Wx*^*mw*^) (**A**) Plant morphology during filling stage. (**B**) Endosperm appearance at different moisture contents. (**C**) Amylose content (AC). (**D**) Gel consistency (GC). (**E**) Taste value of cooked rice. (**F**) Rapid viscosity analysis (RVA) profiles of rice flours. Values with different lowercase letters are significantly different (*P* < 0.05, Student's *t*‐test). Error bars indicate *SD*, *n* = 3.

To evaluate the potential of this rare *Wx* ^*mw*^ allele in improving grain quality of modern high‐yield cultivars, we introgressed *Wx* ^*mw*^ from NIP(*Wx* ^*mw*^) into the local high‐yielding *japonica* rice cultivar 2661 (carrying the *Wx* ^*b*^ allele) by repetitive backcrossing. We designed an allele‐specific DNA marker based on the A/C SNP in exon 6 (Ex 6‐62) for molecular marker‐assisted selection (MAS) (Figure S3). We selected one NIL 2661(*Wx* ^*mw*^) from the BC_6_F_3_ population, and observed no significant differences for the main agronomic traits of 2661(*Wx* ^*mw*^) and its recurrent 2661(*Wx* ^*b*^) parent (Figure 5A). Importantly, 2661(*Wx* ^*mw*^) mature grains exhibited a relatively good transparency, even when their moisture content decreased to about 11.4% (Figure 5B). In terms of grain quality, milled rice from 2661(*Wx* ^*mw*^) showed a significantly lower AC, softer GC and better sensory property than 2661(*Wx* ^*b*^) (Figure 5C–E; Table S4). In addition, rice flours from 2661(*Wx* ^*mw*^) yielded a significantly better RVA curve pattern, that is a significantly higher breakdown, and lower setback than those from 2661(*Wx* ^*b*^) (Figure 5F; Table S5), which is consistent with the hypothesis that rice with good ECQ usually has high breakdown and low setback values (Pang et al., 2016).

## DISCUSSION

### Natural variation and modification of the *Wx* locus

Starch synthesis‐related genes play essential roles in controlling rice ECQ, and *Wx* is the most important gene affecting this trait (Tian et al., 2009). The ever‐increasing demand for rice varieties with high ECQ is driving research toward the identification of natural *Wx* alleles and/or artificially modifying *Wx* to meet the requirements of breeding programs. To date, most of the observed diversity in AC can be linked back to natural allelic variation at the *Wx* locus. Over the past four decades, at least eight *Wx* alleles have been identified (Hoai et al., 2014; Zhang et al., 2019a). In this study, we described a rare *Wx* allele, designated *Wx* ^*mw*^, that fine‐tuned AC by exhibiting moderate GBSSI activity. *Wx* ^*mw*^ is a promising target for improving either ECQ or grain transparency in rice breeding programs, especially for temperate *japonica* rice.

Among SNPs from various *Wx* alleles, the G/T SNP at the splicing donor site of the first intron (Int 1‐1) is considered to be the key factor underlying the observed variation in AC (Wang et al., 1995). This Int 1‐1 SNP can, alone or in combination with other SNPs or InDels (Figure 1E), explain the function associated with available *Wx* alleles (Wanchana et al., 2003; Zhang et al., 2019a). For example, the *Wx* ^*b*^ allele is a combination of T at Int 1‐1 and A at Ex 6‐62 (*Wx* exon 6 SNP) and is strongly associated with low AC (15%–18%) accessions (Wang et al., 1995; Isshiki et al., 1998). Similarly, the *Wx* ^*in*^ allele consists of G at Int 1‐1 and C at Ex 6‐62 and is highly represented among intermediate AC (18%–22%) accessions (Mikami et al., 2008). The newly *Wx* ^*mw*^ allele cloned in this study appears to combine T at Int 1‐1, as in *Wx* ^*b*^, and C at Ex 6‐62, as in *Wx* ^*in*^. Separately, these two SNPs result in a decrease in *Wx* expression or GBSSI activity (Wang et al., 1995; Larkin and Park, 2003); it is therefore satisfying to see that their combination in the recombined *Wx* ^*mw*^ allele also reduces GBSSI activity and thus leads to decreased AC relative to the *Wx* ^*b*^ allele.

*Wx* has been modified by conventional mutagenesis and biotechnological methods such as RNA interference (RNAi) or gene editing by clustered regularly interspaced short palindromic repeats (CRISPR) and CRISPR‐associated nuclease Cas9 (Liu et al., 2003; Ma et al., 2015; Lin et al., 2017). For instance, the *Wx* ^*mq*^ and *Wx* ^*mp*^ alleles were generated by N‐methyl‐N‐nitrosourea‐induced mutagenesis (Sato et al., 2002; Yang et al., 2013). Another 13 *Wx* mutants were induced by treatment with sodium azide, yielding mutant grains with typically opaque endosperm and very low AC ranging from 1.5% to 7.1% (Lin et al., 2017). In terms of the modulation of GBSSI, our previous studies showed that GBSSI activity is very sensitive to amino acid changes (Liu et al., 2014; Zhang et al., 2017), and thus, it is very difficult to generate mutants with moderate AC by editing the *Wx* coding sequence directly. Recent studies have generated a series of glutinous rice mutants by editing the *Wx* coding sequence using CRISPR/Cas9 (Ma et al., 2015; Zhang et al., 2018). Likewise, we have created a series of rice lines with fine‐turned AC levels by editing the *Wx* promoter core regions via CRISPR/Cas9 editing (Huang et al., 2020).

### Relationship between AC and endosperm transparency

Several other regulators can also regulate *Wx* expression at the transcriptional level and thus generate rice grains with low AC but opaque or chalky endosperm. These regulators include dull endosperm 1 (Du1, Zeng et al., 2007), Du3 (Isshiki et al., 2008), quantitative AC (qAC2, Takemoto‐Kuno et al., 2015), floury endosperm 2 (Flo2, Wu et al., 2015), and nuclear factor YB1 (*Os*NF‐YB1, Bello et al., 2019). Rice grain transparency is an important contributing index to the measure of rice quality that affects rice appearance and commercial quality (Zhou et al., 2020). Previous studies have determined that irregular, small and loosely packed starch granules are the main causes behind rice floury or chalky endosperm (Wu et al., 2015; Zhang et al., 2017; Chen et al., 2020). Regarding opaque endosperm, such as in the low AC rice Haopi (carrying the *Wx* ^*op*^ */* ^*hp*^ allele), SEM results indicated that grains presented small empty spaces between starch granules, as well as small holes at the center of starch granules that might be associated with reduced grain transparency (Liu et al., 2009). However, the relationship between AC and grain transparency is unclear. We recently generated several low AC rice lines with dull endosperm by modifying GBSSI activity and established that holes inside starch granules were responsible for the dull appearance of rice grains (Zhang et al., 2017, 2019b). In this study, we observed a similar dullness, especially in the *Wx* ^*mp*^ endosperm, and further demonstrated that this dull grain appearance was negatively correlated with AC from the analysis of NILs and transgenic rice lines carrying various *Wx* alleles (Figures 4, S4). Finally, we established that the hole size within the core of starch granules increased as moisture content decreased and adversely affected the opaque appearance (Figure 4).

Starch granules consist of two different glucose polymers: amylose and amylopectin. Amylose molecules appear to disperse among amylopectin molecules and locate primarily in the amorphous zones in a random coiled form. Previous work on starch granules in cereals has shown that amylose is mainly in the centers (cores) of the granules (Glaring et al., 2006; Cai et al., 2014). Our recent studies indicated that grain transparency was positively correlated with AC, while negatively correlated with the area of cavity within starch granules (Zhang et al., 2017, 2019b). Considering that even a glutenous rice grain can have a transparent phenotype when the moisture content is sufficiently high (>18%), we predicted that AC plays a key role in endosperm appearance and may form a stable and well‐distributed structure with both water and amylopectin (Zhang et al., 2019b). Once the AC drops below a given threshold (<14%), the starch granule structure will become unstable and its semi‐crystalline structure may retract in the center of the starch granule, resulting in the appearance of holes. Indeed, we recently discovered that amylopectin may function together with amylose to form a stable semi‐crystalline structure and a transparent endosperm in transgenic RNAi rice lines against *soluble starch synthase II‐2* (*SSSII‐2*), even with extremely low AC (Li et al., 2018).

### Use of *Wx* ^*mw*^ to improve rice grain quality by MAS

Rice cultivars with varied AC values are an agronomic necessity to accommodate the preferences of different countries and regions (Custodio et al., 2019; Zhou et al., 2020). Currently, soft rice with low AC is preferred in some regions in China, where soft *indica* cultivars usually carry the rare *Wx* ^*op/hp*^ allele (Larkin and Park, 2003; Liu et al., 2009) and soft *japonica* cultivars carry the *Wx* ^*mp*^ allele, which was generated through artificial induction (Yang et al., 2013). However, all soft rice cultivars bearing these alleles usually also display a dark or dull endosperm appearance and are sometimes too sticky after cooking. Our data demonstrated that *japonica* rice with the *Wx* ^*mw*^ allele had a higher AC than either *Wx* ^*op/hp*^ or *Wx* ^*mp*^. In addition, the presence of the *Wx* ^*mw*^ allele improved endosperm transparency. Moreover, the taste of the new *Wx* ^*mw*^ rice lines generated here improved greatly when compared to other cultivars such as NIP and 2611. Therefore, the newly identified *Wx* ^*mw*^ allele may constitute a useful genetic resource to improve ECQ and endosperm appearance, especially in temperate *japonica* rice cultivars, to meet the demands of consumers in specific regions.

Marker‐assisted selection has been instrumental in addressing the limitations of AC measurements and producing improved super‐rice with high yield and superior quality (Zeng et al., 2017). In this study, we developed an allele‐specific DNA marker based on the A/C SNP in exon 6 and generated two NILs in two distinct backgrounds (NIP and 2661) with the help of MAS. The cultivar 2661 is high‐yielding, and the derived NIL showed the same improved ECQ and grain appearance as when *Wx* ^*mw*^ was introgressed into the NIP background, which underscores the advantages of the *Wx* ^*mw*^ allele for improving rice grain quality. The use of SNP markers based on Kompetitive Allele‐Specific PCR (KASP) has recently been shown to be suitable for high‐throughput automated genotyping for rice breeding, and functional SNPs between different *Wx* alleles can be used for the development of KASP markers (Yang et al., 2019). The molecular marker RM190 can be used to distinguish between *Wx* ^*in*^ and *Wx* ^*b*^ alleles in some rice cultivars (Biselli et al., 2014). Moreover, RM190 can be used to distinguish between *Wx* ^*mw*^ and *Wx* ^*a*^ in some cultivars. Thus, both RM190 and allele‐specific DNA markers can be used in rice breeding, depending on the *Wx* allele polymorphism between different rice cultivars.

## CONCLUSION

The AC of endosperm starch is the key factor determining rice grain quality, especially ECQ and grain transparency. Of the many genes involved in starch biosynthesis in the rice endosperm, *Wx* is largely responsible for AC, and thus ECQ (Tian et al., 2009; Zhang et al., 2019a). The major goal of rice grain quality breeding programs is not only good ECQ but also transparent endosperm appearance. Here, we cloned *Wx* ^*mw*^, a rare *Wx* allele responsible for a favorable AC, improved ECQ and grain transparency. Notably, we successfully harnessed the *Wx* ^*mw*^ allele to breed new rice cultivars with moderate AC and good grain appearance via MAS. Our results suggest that *Wx* ^*mw*^ is a promising allele for grain quality improvement in rice breeding programs, especially for improving ECQ and grain transparency in high‐yielding *japonica* cultivars.

## MATERIALS AND METHODS

### Plant materials and growth conditions

We used a number of rice (*Oryza sativa*) accessions in this study. These accessions included a set of six varieties with different *Wx* alleles and AC for comparison of grain qualities (Figure S1; Table S8): the tropical *japonica* landrace MWG, carrying the novel *Wx* ^*mw*^ allele; the three temperate *japonica* cultivars NIP, carrying the *Wx* ^*b*^ allele, Guanglingxiangnuo (GLXN, carrying the *wx* allele), and Nangeng 46 (NG46, carrying the *Wx* ^*mp*^ allele); and the two *indica* cultivars IR64 (carrying the *Wx* ^*in*^ allele) and Teqing (TQ, carrying the *Wx* ^*a*^ allele). We also worked with the other temperate *japonica* cultivar 2661 (carrying the *Wx* ^*b*^ allele) and two NILs, NIP(*wx*) and NIP(*Wx* ^*mp*^), for functional analyses. The NILs NIP(*wx*) and NIP(*Wx* ^*mp*^) were generated by introgression of the *wx* and *Wx* ^*mp*^ alleles, respectively, in the NIP background as described previously (Zhang et al., 2019a). We also used a panel of 370 worldwide rice accessions (including the above rice varieties) (Table S8) from our previous study (Zhang et al., 2019a). We cultivated all rice plants under natural field conditions at experimental stations located in Yangzhou (Jiangsu Province, 32°23′N). For analysis of rice phenotypes and grain quality, we planted the NILs in triplicate during the summer season in Yangzhou, and harvested seeds at maturity from 20 plants in the middle of each plot before allowing the seeds to air‐dry. For gene expression and GBSSI protein assays, we collected rice seeds at different development stages from three biological replicates.

### Construction of the mapping population and near‐isogenic lines (NILs)

For genetic analysis and fine mapping of the candidate gene for low AC in the MWG background, we generated an F_2_ population by crossing MWG and NIP. For gene functional analyses, we used the NIL NIP(*wx*) as the recipient for transformation. For gene expression and grain quality analyses, we generated the NIL NIP(*Wx* ^*mw*^) in the NIP background by introducing the *Wx* ^*mw*^ allele from the donor MWG, followed by seven rounds of backcrossing, assisted with the molecular markers listed below (Figure S3A). To evaluate the effects of *Wx* ^*mw*^ on breeding programs, we introgressed the *Wx* ^*mw*^ allele into the *japonica* cultivar 2661 (with a high yield potential, originating from Jiangsu Province) followed by six rounds of backcrossing to 2661, yielding the new NIL 2661(*Wx* ^*mw*^) (Figure 5). We planted all rice materials mentioned above during the summer season in Yangzhou (Jiangsu, China, 32°23′N) or during the winter season in Lingshui (Hainan, China, 18°30′N).

### DNA extraction and molecular marker development

We extracted rice genomic DNA from young leaves with the cetyltrimethylammonium bromide method (Murray and Thompson, 1980). For molecular marker analyses, we performed polymerase chain reaction (PCR) as described previously (Zhang et al., 2011). For gene mapping and cloning, we used published molecular markers (Zhang et al., 2011) or designed new markers with Vector NTI software (Lu and Moriyama, 2004) based on sequence polymorphisms between the cultivars *japonica* NIP and *indica* 9311 (Table S1). For the construction of the NIL NIP(*Wx* ^*mp*^), we developed the two allele‐specific PCR (AS‐PCR) markers WXmp1/2 and used for MAS, as reported by (Yang et al., 2013). For construction of NILs NIP(*Wx* ^*mw*^) and 2661(*Wx* ^*mw*^), we developed the two AS‐PCR markers WXmw1/2 to detect the A‐to‐C mutation in exon 6 of *Wx* (Table S1).

### Genotyping, sequencing, and population analyses

To determine the genetic background of each NIL, we used 120 molecular markers distributed over the 12 rice chromosomes, as described previously (Zhang et al., 2011). We amplified the *Wx* locus by PCR using three primer pairs (Table S1) from total genomic DNA extracted from NIP or MWG. We sequenced the PCR products and aligned their sequences using the software Dnastar 7.1 (DNASTAR Inc., USA). We conducted a multiple sequence alignment using ClustalX (Larkin et al., 2007). For bioinformatics analyses, we made use of *Wx* genomic sequences (5 318 bp) from 370 rice accessions (Table S8). We identified the various *Wx* allelic types found in these accessions according to our previous study (Zhang et al., 2019). We carried out phylogenetic and molecular evolutionary analyses using MEGA version X (Kumar et al., 2018).

### Plasmids and rice transformation

For functional complementation analysis, we PCR‐amplified a 8 kbp genomic fragment covering the entire *Wx* locus with primers WxG1 and WxG2 (Table S1) from NIP, MWG, and NG46, respectively. Polymerase chain reactions were carried out in a 50 µL reaction volume using PrimeSTAR® HS DNA Polymerase (TaKaRa, Kyoto, Japan). Amplification conditions consisted of 35 cycles at 98°C for 10 s, 4 min at 68°C, followed by 2 min at 72°C. We cloned the resulting PCR products into the pCAMBIA1300 vector at the *Bam*HI and *Kpn*I sites. We introduced the constructs p1300‐*Wx* ^*b*^ *,* p1300‐*Wx* ^*mw*^, and p1300‐*Wx* ^*mp*^ (Figure S2A) into Agrobacterium (*Agrobacterium tumefaciens*) strain EHA105 and transformed the glutinous NIL NIP(*wx*) via Agrobacterium‐mediated transformation (Liu et al., 1998). We determined transgene copy number in transgenic rice with the SYBR Green Real‐Time PCR Kit (TaKaRa, TB Green™ Premix Ex Taq™ GC) and a real‐time PCR detection system (Bio‐Rad CFX96), as described by (Ding et al., 2004). For real‐time quantitative PCR, we employed 10 ng of genomic DNA as template, and amplified the DNA samples with two independent primer sets specific for *Wx* and an endogenous single‐copy molecular marker (R10M10; Table S1).

### RNA extraction and real‐time quantitative PCR analysis

We extracted total RNA from developing seeds at 5, 10, 15, 20, and 25 d after flowering (DAF) with the RNAprep pure Plant Kit (Tiangen, Beijing), according to the manufacturer's protocol. We synthesized first‐strand complementary DNAs (cDNAs) using the PrimeScript™ RT reagent Kit (TaKaRa), and performed real‐time quantitative PCR analysis with the SYBR Green real‐time quantitative PCR method. We performed three technical replicates per template and with three biological replicates for each sample to obtain an average value for relative expression levels. *OsACTIN1* was used as the internal control. All primer sets are listed in Table S1. We analyzed results using the 2^–ΔΔCt^ method (Livak and Schmittgen, 2001).

### Assay of GBSSI protein and activity

We collected developing seeds at 5, 10, 15, 20, and 25 DAF, and then ground them to powder in liquid nitrogen after removing husks and embryos. We used the resulting powders for GBSSI activity assay as described previously (Liu et al., 2014). For total seed protein analysis, we took a 100 mg aliquot of the powder from 20 DAF seeds for protein extraction, as described by (Yamagata et al., 1982). Moreover, we extracted starch granule‐bound GBSSI as described by (Liu et al., 2014), followed by separation on sodium dodecyl sulfate‐polyacrylamide gel electrophoresis gels, according to standard procedures.

### Grain quality analyses

For grain transparency analysis, we harvested mature seeds and dried them in a drying oven (40 °C) for 2, 4, 6, 8, 12, and 24 h before milling to white rice. We measured the moisture content of milled grains using a halogen moisture analyzer (Mettler Toledo MJ33, Switzerland). For grain physicochemical quality analyses, we air‐dried and milled mature seeds to white rice. We ground a fraction of the polished rice grains into flour and used this for starch isolation, as described previously (Zhang et al., 2013). To obtain grain cross‐sections, we randomly selected five rice grains for each line, froze them in liquid nitrogen, broke them by hand, and then observed the resulting sections using an environmental SEM (Philips XL‐30). We determined the AC of the rice flours using the iodine colorimetric method (Tan et al., 1999). We measured gel consistency (GC) according to the method of (Tan et al., 1999), and GT according to (Zhang et al., 2013) with a DSC 200 F3 (Netzsch Instruments NA LLC, Burlington, MA, USA). We investigated the pasting properties of the rice flours with a Rapid Visco‐Analyzer (Techmaster, Newport Scientific, Warriewood, Australia) as reported by (Zhang et al., 2013). The taste value of cooked rice was determined with an RCTA‐11A Taste Analyzer (Satake, Japan) as described previously (Li et al., 2018).

### Statistical analysis

For sample characterization, we performed three replicate measurements, unless otherwise specified. All data represent the mean ± *SD*. We subjected all data to one‐way analysis of variance and Student's *t*‐test analysis, using the SPSS 16.0 statistical software program. Results with a probability value of *P* < 0.05 were considered statistically significant.

## AUTHOR CONTRIBUTIONS

Q.Q.L., C.Z., and M.G. conceived and designed the experiments. C.Z., Y.Y., Y.L., L.Z., and S.T. performed the experiments. L.Z., S.C., J.Z., and X.L. analyzed the data. Q.F.L. and X.F. prepared the seed samples. Z.C. and Q.Q.L. wrote the manuscript. All authors have read and approved of the manuscript.

## Supporting information

Additional Supporting Information may be found online in the supporting information tab for this article: http://onlinelibrary.wiley.com/doi/10.1111/jipb.13010/suppinfo

**Figure S1.** Grain physicochemical characteristics of six rice varieties carrying different *Wx* alleles
Grain appearance and physicochemical characteristics of six rice varieties carrying different *Wx* alleles. **(A)** Gelatinization temperature (GT) of rice flours. **(B)** Rapid viscosity analysis (RVA) profiles of purified rice starches from mature grains. Rice varieties: tropical *japonica* landrace Mowanggu (MWG); three temperate *japonica* cultivars Nipponbare (NIP), Guanglingxiangnuo (GLXN) and Nangeng 46 (NG46); two *indica* cultivars IR64 and Teqing (TQ). Values labelled with different lowercase letters are significantly different by one‐way ANOVA with multiple comparisons (*p* < 0.05). The error bars indicate standard deviation (*SD*).
**Figure S2.** T‐DNA structure of the constructs used for rice transformation and PCR verification of transgenic rice
**(A)** T‐DNA structure of the constructs used for rice transformation. The intact *Wxb*, *Wxmw* or *Wxmp* genomic fragments inserted between *Hind*III and *Kpn*I in the T‐DNA region are derived from NIP, MWG or NG46, respectively. The indicated “T‐G(A)‐A(C)” in the *Wx* coding region corresponds to polymorphic nucleotides from the single nucleotide polymorphism (SNP) Int1‐1, Ex4‐53 and Ex6‐62, respectively, as shown in Figure 1F. ATG and TGA represent the start and stop codons of *Wx*, respectively; RB and LB indicate right and left borders, respectively; 35S and 35S polyA mean the promoter and polyA sequences of the cauliflower Mosaic Virus (CaMV) 35S gene, respectively. **(B)** PCR verification on transgenic rice plants. We amplified the hygromycin resistance gene to validate transgenic plants. WT: receptor parent NIP(*wx*). NIP(*wx*)‐*Wxb*, NIP(*wx*)‐*Wxmw* and NIP(*wx*)‐*Wxmp* transformants carrying the *Wxb*, *Wxmw* or *Wxmp* transgene in the NIP(*wx*) background, respectively.
**Figure S3.** Construction of near isogenic lines (NILs) carrying different *Wx* alleles and phenotypic and allele identification of NILs in the *japonica* NIP background
**(A)** Construction of near isogenic lines (NILs) carrying different *Wx* alleles. MAS, molecular marker‐assisted selection. **(B)** Phenotypic and **(C, D)** allele identification of NILs in the *japonica* NIP background. **(B)** Plant morphology at maturity. **(C, D)** Allele‐specific primer PCR (AS‐PCR) for the detection of *Wxmw* **(C)** and *Wxmp* **(D)**, respectively. NIP or NIP(*Wxb*) represents the *japonica* cultivar Nipponbare as the recurrent parent carrying *Wxb* allele, while MWG and NG46 are the donors with the *Wxmw* or *Wxmp* allele, respectively.
**Figure S4.** Endosperm appearance of milled rice and morphology of grain transverse sections of the glutinous line NIP(*wx*) and transgenic derivatives by scanning electron microscopy
**(A)** Milled rice under dry conditions. **(B–D)** Transverse sections of mature grains from the glutinous line NIP(*wx*). **(E–G)** 31 Transverse sections of mature grains from NIP(*wx*)‐*Wxb* transformants. **(H–J)** Transverse sections of mature grains from NIP(*wx*)‐*Wxmw* transformants. **(K–M)** Transverse sections of mature grains from NIP(*wx*)‐*Wxmp* transformants. Red arrows indicate holes within starch granules.
**Table S1.** Primers used in this study
**Table S2.** Pasting properties of flours from different rice varieties
Data represent means ± standard deviations, *n* = 2. For each column in the same flour or starch samples, values displaying different lowercase letters are significantly different by one‐way ANOVA with multiple comparisons (*p* < 0.05). Rice varieties: tropical *japonica* landrace Mowanggu (MWG); three temperate *japonica* cultivars Nipponbare (NIP), Guanglingxiangnuo (GLXN) and Nangeng 46 (NG46); two *indica* cultivars IR64 and Teqing (TQ).
**Table S3.** Thermal properties of mature rice grains from transgenic rice and its wild type
The data represent means ± standard deviation, n = 3. *T*o, *Tp*, *Tc*, and Δ*H* indicate onset temperature, peak temperature, conclusion temperature, and enthalpy of gelatinization, respectively. Means with different lowercase letters in each column for the same cultivar are significantly different by one‐way ANOVA with multiple comparisons (*p* < 0.05).
**Table S4.** Taste value and physiochemical properties of the mature grains from NILs carrying different *Wx* alleles
Data represent means ± standard deviation, n = 3. *T*o, *Tp*, *Tc* and Δ*H* indicate onset temperature, peak temperature, conclusion temperature, and enthalpy of gelatinization, respectively. Means with different lowercase letters in each column for the same cultivar are significantly different by one‐way ANOVA with multiple comparisons and student's *t*‐test analysis (*p* < 0.05).
**Table S5.** Pasting properties of rice flours from near‐isogenic lines (NILs) in the Nipponbare (NIP) and 2661 backgrounds
Data represent means ± standard deviation, *n* = 2. For each column in the same cultivar background, values with different lowercase letters are significantly different by one‐way ANOVA with multiple comparisons and student's *t*‐test analysis (*p* < 0.05).
**Table S6.** Average genetic distances between groups carrying various *Wx* alleles
Population average distances were calculated from SNPs in 370 accessions (Table S8) using the bootstrap method.
**Table S7.** Moisture content of rice grains from different NILs after gradient drying as shown in Figure 3A
The mature seeds were dried directly in a drying oven (40°C) for 2, 4, 6, 8, 12, and 24 h and then milled to white rice for measurement of moisture content. Data represent means ± standard deviations, n = 2. For each column, values with different lowercase letters are significantly different by one‐way ANOVA with multiple comparisons (*p* < 0.05).
**Table S8.** List of rice accessions used in this study

Supporting information.
