# Supplementary material for: A rare Waxy allele coordinately improves rice eating and cooking quality and grain transparency
Source: J Integr Plant Biol. 2020 Dec 29;63(5):889–901. doi: 10.1111/jipb.13010 (PMC8246539; doi:10.1111/jipb.13010)
Supplement: Supplementary file 1 — Figure S1. Grain physicochemical characteristics of six rice varieties carrying different Wx alleles Grain appearance and physicochemical characteristics of six rice varieties carrying different Wx alleles. (A) Gelatinization temperature (GT) of rice flours. (B) Rapid viscosity analysis (RVA) profiles of purified rice starches from mature grains. Rice varieties: tropical japonica landrace Mowanggu (MWG); three temperate japonica cultivars Nipponbare (NIP), Guanglingxiangnuo (GLXN) and Nangeng 46 (NG46); two indica cultivars IR64 and Teqing (TQ). Values labelled with different lowercase letters are significantly different by one‐way ANOVA with multiple comparisons (p < 0.05). The error bars indicate standard deviation (SD). Figure S2. T‐DNA structure of the constructs used for rice transformation and PCR verification of transgenic rice (A) T‐DNA structure of the constructs used for rice transformation. The intact Wxb, Wxmw or Wxmp genomic fragments inserted between HindIII and KpnI in the T‐DNA region are derived from NIP, MWG or NG46, respectively. The indicated “T‐G(A)‐A(C)” in the Wx coding region corresponds to polymorphic nucleotides from the single nucleotide polymorphism (SNP) Int1‐1, Ex4‐53 and Ex6‐62, respectively, as shown in Figure 1F. ATG and TGA represent the start and stop codons of Wx, respectively; RB and LB indicate right and left borders, respectively; 35S and 35S polyA mean the promoter and polyA sequences of the cauliflower Mosaic Virus (CaMV) 35S gene, respectively. (B) PCR verification on transgenic rice plants. We amplified the hygromycin resistance gene to validate transgenic plants. WT: receptor parent NIP(wx). NIP(wx)‐Wxb, NIP(wx)‐Wxmw and NIP(wx)‐Wxmp transformants carrying the Wxb, Wxmw or Wxmp transgene in the NIP(wx) background, respectively. Figure S3. Construction of near isogenic lines (NILs) carrying different Wx alleles and phenotypic and allele identification of NILs in the japonica NIP background (A) Construction of near isogenic [file JIPB-63-889-s001.pdf]

A rare *Waxy* allele coordinately improves rice eating and cooking quality and grain transparency

Changquan Zhang[1,2], Yong Yang[1], Shengjie Chen[2, 3], Xueju Liu[2], Jihui Zhu[1,2], Lihui Zhou[2], Yan Lu[2], Qianfeng Li[1,2], Xiaolei Fan[1,2], Shuzhu Tang[1,2], Minghong Gu[1,2], and Qiaoquan Liu[1,2]*

SUPPORTING INFORMATION

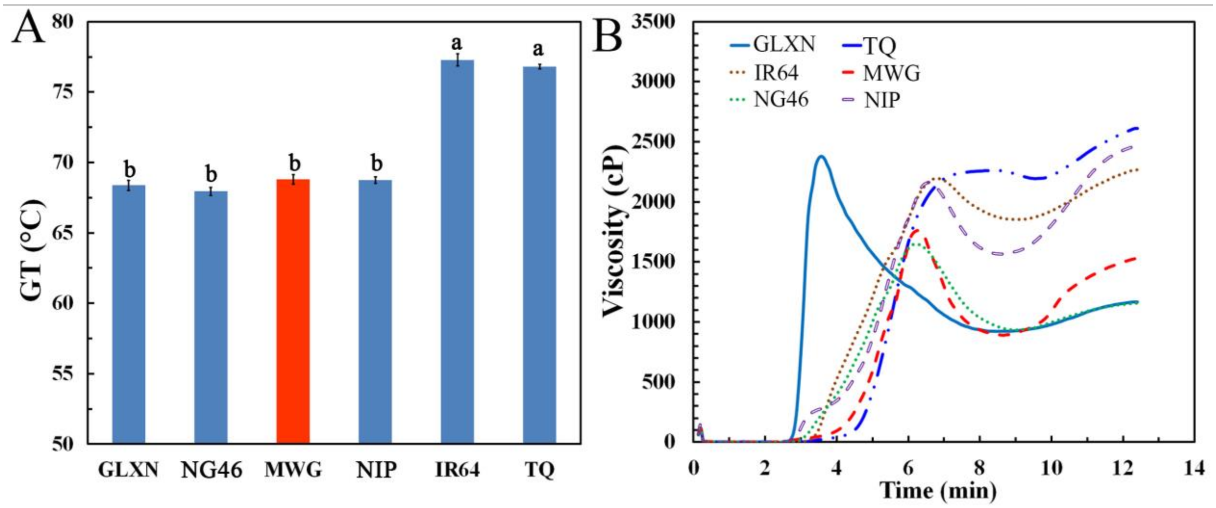

Figure S1. Grain physicochemical characteristics of six rice varieties carrying different *Wx* alleles

Grain appearance and physicochemical characteristics of six rice varieties carrying different *Wx* alleles. **(A)** Gelatinization temperature (GT) of rice flours. **(B)** Rapid viscosity analysis (RVA) profiles of purified rice starches from mature grains. Rice varieties: tropical *japonica* landrace Mowanggu (MWG); three temperate *japonica* cultivars Nipponbare (NIP), Guanglingxiangnuo (GLXN) and Nangeng 46 (NG46); two *indica* cultivars IR64 and Teqing (TQ). Values labelled with different lowercase letters are significantly different by one-way ANOVA with multiple comparisons ($p < 0.05$). The error bars indicate standard deviation (SD).

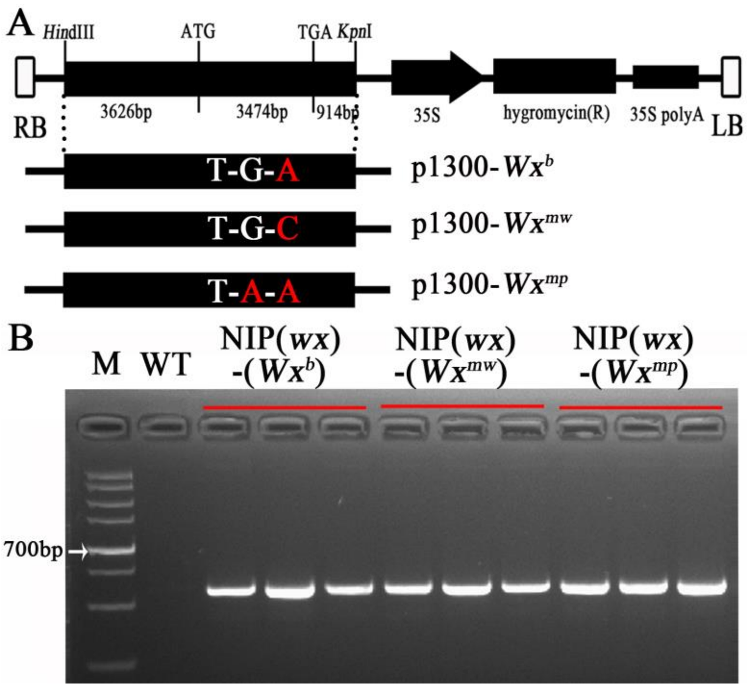

Figure S2. T-DNA structure of the constructs used for rice transformation and PCR verification of transgenic rice

(A) T-DNA structure of the constructs used for rice transformation. The intact *Wx*^{b}, *Wx*^{mw} or *Wx*^{mp} genomic fragments inserted between *Hind*III and *Kpn*I in the T-DNA region are derived from NIP, MWG or NG46, respectively. The indicated "T-G(A)-A(C)" in the *Wx* coding region corresponds to polymorphic nucleotides from the single nucleotide polymorphism (SNP) Int1-1, Ex4-53 and Ex6-62, respectively, as shown in Figure 1F. ATG and TGA represent the start and stop codons of *Wx*, respectively; RB and LB indicate right and left borders, respectively; 35S and 35S polyA mean the promoter and polyA sequences of the cauliflower Mosaic Virus (CaMV) 35S gene, respectively. **(B)** PCR verification on transgenic rice plants. We amplified the hygromycin resistance gene to validate transgenic plants. WT: receptor parent NIP(*wx*). NIP(*wx*)-*Wx*^{b}, NIP(*wx*)-*Wx*^{mw} and NIP(*wx*)-*Wx*^{mp} transformants carrying the *Wx*^{b}, *Wx*^{mw} or *Wx*^{mp} transgene in the NIP(*wx*) background, respectively.

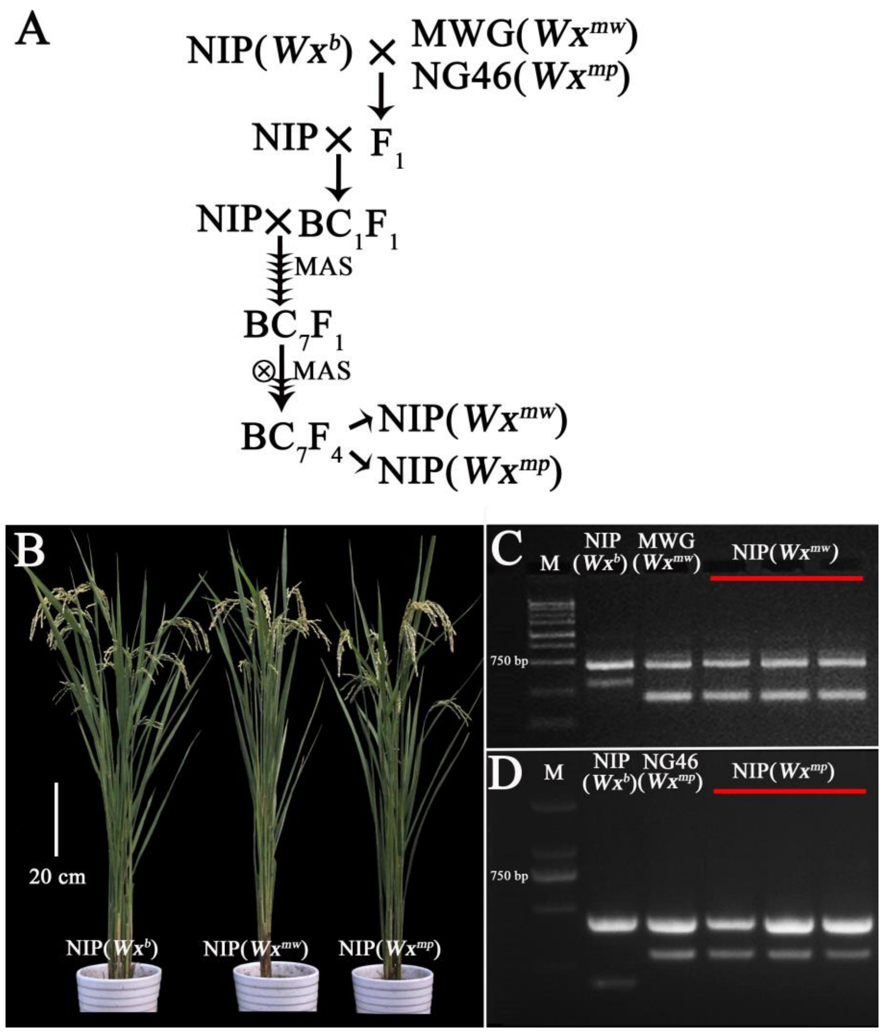

Figure S3. Construction of near isogenic lines (NILs) carrying different *Wx* alleles and phenotypic and allele identification of NILs in the *japonica* NIP background

(A) Construction of near isogenic lines (NILs) carrying different *Wx* alleles. MAS, molecular marker-assisted selection. **(B)** Phenotypic and **(C, D)** allele identification of NILs in the *japonica* NIP background. **(B)** Plant morphology at maturity. **(C, D)** Allele-specific primer PCR (AS-PCR) for the detection of Wx^{mw} **(C)** and Wx^{mp} **(D)**, respectively. NIP or NIP(Wx^b) represents the *japonica* cultivar Nipponbare as the recurrent parent carrying Wx^b allele, while MWG and NG46 are the donors with the Wx^{mw} or Wx^{mp} allele, respectively.

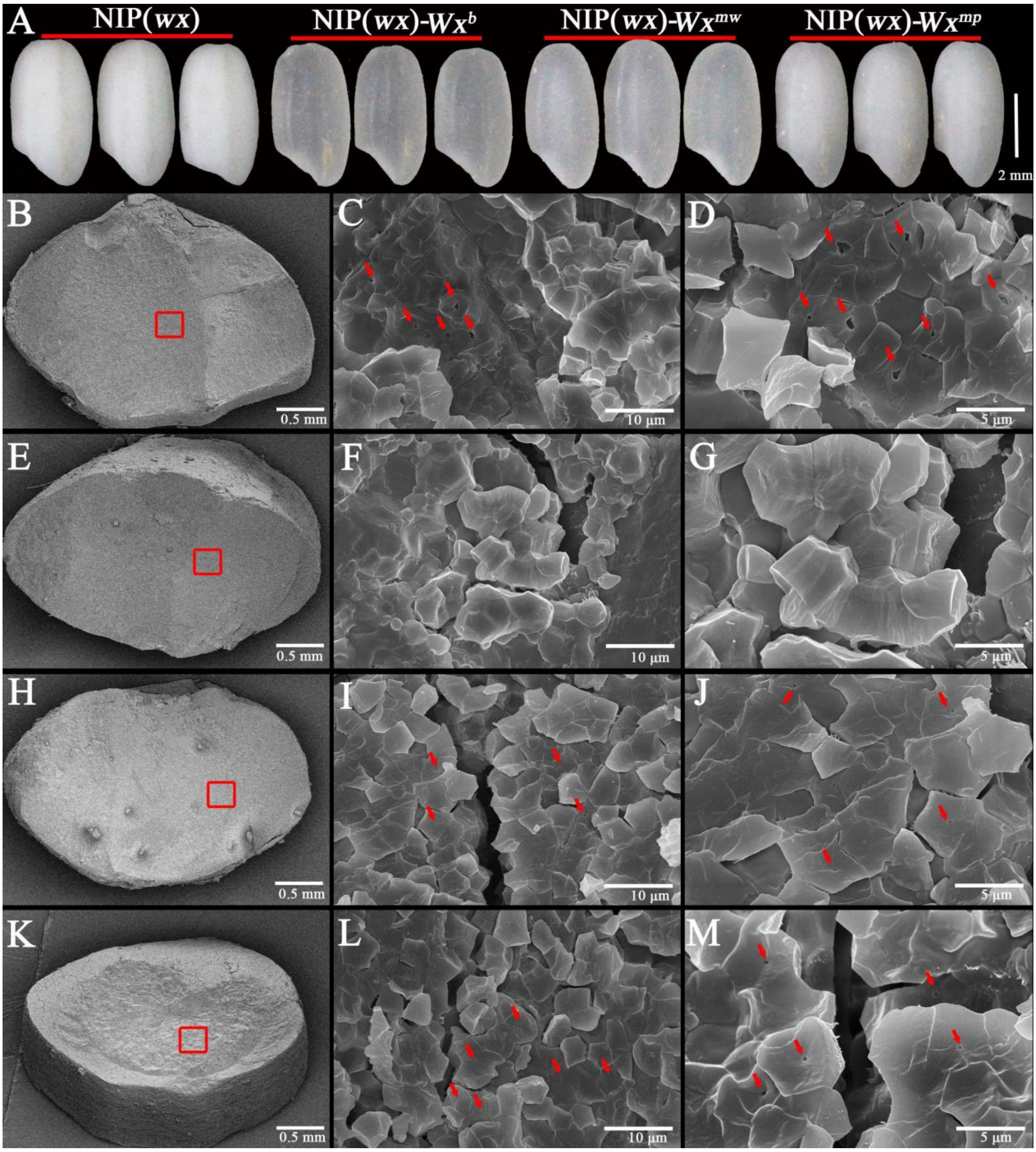

Figure S4. Endosperm appearance of milled rice and morphology of grain transverse sections of the glutinous line NIP(*wx*) and transgenic derivatives by scanning electron microscopy

(A) Milled rice under dry conditions. **(B–D)** Transverse sections of mature grains from the glutinous line NIP(*wx*). **(E–G)** 31 Transverse sections of mature grains from NIP(*wx*)-Wx^{b} transformants. **(H–J)** Transverse sections of mature grains from NIP(*wx*)-Wx^{mw} transformants. **(K–M)** Transverse sections of mature grains from NIP(*wx*)-Wx^{mp} transformants. Red arrows indicate holes within starch granules.

Table S1. Primers used in this study

Name	Primers (5'-3')	Purpose
Chr601	F: GTCCATCCAAGAACATAACAACAA<br>R: CAAGGTTAGGTACATTTTACAGCG	Gene mapping
RM586	F: ACCTCGCGTTATTAGGTACCC<br>R: GAGATACGCCAACGAGATACC	
CHR608	F: ACACGTACGGTCGATCGGAA<br>R: GCTTAATGGTGTAGCTAGATCGAGA	
Chr613	F: AATGACGATGAGGTTGAAAAGGTA<br>R: ATCACACAGGGATATTGCACA	
Chr602	F: CCTTGGCCCTTGAATAGTTG<br>R: TGCTCGATTCTGACAAAGTT	
RM276	F: CTCAACGTTGACACCTCGTG<br>R: TCCTCCATCGAGCAGTATCA	
CS0610	F: GCACGATGGAGAAATGGA<br>R: ACAAGACAACGGTGGAGT	
CS0615	F: ATTTTCCAATCGTCTAAG<br>R: GAATCGTACCCAAGTTTT	
RM454	F: CTCAAGCTTAGCTGCTGCTG<br>R: GTGATCAGTGCACCATAGCG	
CS0628	F: GCGTCGGACTTACGTGAGAAGG<br>R: CTCAGCATGGAGACGAGAGACG	
WXGf	F: TAAGCTTGGACGAGTTGGGTGAACCTGGGA<br>R: CGCCTGCAAAGAACACAAGAACACAACATT	Gene amplification, sequencing and construction of transgenic vectors
WXGr	F: AACAATTCAATTCAGTGCAGAGATCTTCCACA<br>R: TGGTACCTGAACTTGACGTAGACAGACGTACGATA	
SPS	F: TTGCGCCTGAACGGATAT<br>R: CGGTTGATCTTTTCGGGATG	RT-qPCR detection of *Wx* copy number
WxN	F: AACTGGATGAAGGCCGGAAT<br>R: TGTACTTGTCCTTGCTGGGAT	
WXmp1	F: ATGTTGTGTTCTTGTGTTCTTTGCAGGC<br>R: GTCGATGAACACACGGTCGACTCAAT	Ex 4-53(G/A)
WXmp2	F: GGGTGAGGTTTTTCCATTGCTACAATCG<br>R: GTAGATCTTCTCACCGGTCTTTCCCCAA	
WXmw1	F: AACAACCCATACTTCAAAGGAACATC<br>R: GTAGATGCCATTGGGCTGGTAGT	Ex 6-62(A/C)
WXmw2	F: GCTTAGCTTCCACTGGTGATTTCA<br>R:TCTTGAGATCAATTGTAACTCCCCAT	
WXrt	F: ATTCCTTCAGTTCTTTGTCTATCTCA<br>R: ATGGTGGTTGTCTAGCTGTTGC	RT-qPCR detection
OsActin1	F: CCAAGGCCAATCGTGAGAAGA<br>R: AATCAGTGAGATCACGCCCAG	

Table S2. Pasting properties of flours from different rice varieties

Samples	Varieties	Peak Visco (cP)	Trough Visco (cP)	Breakdown (cP)	Final Visco (cP)	Setback (cP)	Peak Time (min)	Pasting Temp (℃)
	GLXN	1356.50±10.61^{e}	425.00±9.89^{e}	931.50±0.71^{d}	568.00±5.66^{e}	-788.50±4.95^{e}	3.38±0.03^{b}	70.93±0.04^{c}
	NG46	2711.00±18.38^{d}	1836.00±38.18^{d}	875.00±56.57^{e}	2569.50±34.65^{d}	-141.50±16.26^{c}	6.39±0.14^{a}	70.85±0.06^{d}
Flours	MWG	3317.50±10.61^{a}	2046.00±24.04^{b}	1271.50±13.44^{b}	3109.50±16.26^{c}	-212.00±11.31^{d}	6.34±0.06^{a}	72.57±0.04^{a}
	NIP	3122.50±19.09^{b}	1967.50±14.85^{c}	1155.00±4.24^{c}	3163.50±21.92^{c}	41.00±2.83^{b}	6.24±0.01^{a}	72.38±0.03^{c}
	IR64	2874.00±21.21^{c}	2369.50±7.78^{a}	504.50±13.43^{f}	3638.50±13.44^{b}	764.50±7.78^{a}	6.48±0.05^{a}	70.04±0.06^{c}
	TQ	3387.00±26.35^{a}	2072.50±31.13^{b}	1315.5±18.52^{a}	4158.00±26.57^{a}	771.50±21.54^{a}	6.52±0.12^{a}	74.34±0.05^{a}
	GLXN	2347.51±40.31^{a}	909.50±17.68^{d}	1438.00±22.63^{a}	1152.00±21.21^{e}	-1195.50±19.09^{d}	3.62±0.02^{d}	70.10±0.07^{f}
	NG46	1639.50±12.03^{e}	923.00±15.56^{e}	716.50±3.54^{c}	1148.50±10.61^{e}	-491.00±1.41^{e}	6.19±0.01^{c}	76.49±0.08^{e}
Starches	MWG	1781.50±27.58^{d}	900.00±14.14^{d}	881.50±13.43^{b}	1540.00±18.38^{d}	-241.5±9.19^{f}	6.23±0.04^{c}	90.19±0.01^{b}
	NIP	2214.50±27.57^{b}	1862.00±12.73^{c}	352.50±14.85^{d}	2275.50±13.43^{c}	61.00±14.14^{c}	6.78±0.04^{b}	78.85±0.11^{d}
	IR64	2231.50±31.02^{b}	1924.00±21.27^{b}	308.50±19.32^{e}	2315.00±17.35^{b}	84.50±11.24^{b}	6.81±0.03^{b}	82.64±0.12^{c}
	TQ	2184.00±12.73^{c}	2169.00±14.12^{a}	15.00±1.41^{f}	2625.00±19.80^{a}	441.00±32.53^{a}	7.11±0.15^{a}	93.31±0.13^{a}

Data represent means ± standard deviations, $n = 2$. For each column in the same flour or starch samples, values displaying different lowercase letters are significantly different by one-way ANOVA with multiple comparisons ($p < 0.05$). Rice varieties: tropical *japonica* landrace Mowanggu (MWG); three temperate *japonica* cultivars Nipponbare (NIP), Guanglingxiangnuo (GLXN) and Nangeng 46 (NG46); two *indica* cultivars IR64 and Teqing (TQ).

Table S3. Thermal properties of mature rice grains from transgenic rice and its wild type

**Transgenic lines**	**T_o (°C)**	**T_p (°C)**	**T_c (°C)**	**Δ*H* (J•G^{-1})**
NIP(*wx*) (wild type)	65.90±0.10^{c}	68.85±0.05^{c}	75.05±0.05^{c}	10.56±0.68^{a}
NIP(*wx*)-*Wx*^{b}-1#	68.15±0.25^{a}	73.95±0.15^{a}	81.70±0.51^{a}	8.12±0.54^{c}
NIP(*wx*)-*Wx*^{b}-2#	67.97±0.10^{a}	74.55±0.25^{a}	80.40±1.03^{a}	8.03±0.73^{c}
NIP(*wx*)-*Wx*^{b}-3#	68.85±0.05^{a}	72.55±0.05^{a}	81.36±0.21^{a}	8.23±0.22^{c}
NIP(*wx*)-*Wx*^{mw}-1#	66.95±0.15^{b}	71.34±0.21^{b}	76.60±0.10^{b}	8.98±0.56^{b}
NIP(*wx*)-*Wx*^{mw}-2#	66.82±0.21^{b}	71.20±0.08^{b}	77.55±0.25^{b}	9.12±0.38^{b}
NIP(*wx*)-*Wx*^{mw}-3#	66.47±0.09^{b}	70.59±0.21^{b}	76.14±0.73^{b}	9.31±0.71^{b}
NIP(*wx*)-*Wx*^{mp}-1#	66.29±0.14^{b}	69.89±0.35^{b}	76.08±0.08^{b}	9.78±0.23^{b}
NIP(*wx*)-*Wx*^{mp}-2#	66.46±0.52^{b}	70.13±0.11^{b}	76.25±0.31^{b}	9.27±0.09^{b}
NIP(*wx*)-*Wx*^{mp}-3#	66.28±0.82^{b}	69.87±0.63^{b}	76.75±0.43^{b}	9.87±0.26^{b}

The data represent means ± standard deviation, n = 3. T_o, T_p, T_c, and Δ*H* indicate onset temperature, peak temperature, conclusion temperature, and enthalpy of gelatinization, respectively. Means with different lowercase letters in each column for the same cultivar are significantly different by one-way ANOVA with multiple comparisons ($p < 0.05$).

Table S4. Taste value and physiochemical properties of the mature grains from NILs carrying different *Wx* alleles

Lines	Taste value	AC (%)	GC (mm)	T_o (°C)	T_p (°C)	T_c (°C)	ΔH (J•G^{-1})
NIP(Wx^{b})	63.13 ± 0.48^{c}	16.08 ± 0.11^{a}	82.58 ± 2.21^{c}	68.75 ± 0.05^{a}	74.35 ± 0.05^{a}	81.35 ± 0.15^{a}	8.13 ± 0.56^{b}
NIP(Wx^{mw})	68.21 ± 0.56^{b}	13.55 ± 0.09^{b}	91.25 ± 3.10^{b}	66.40 ± 0.30^{b}	72.53 ± 0.17^{b}	78.95 ± 0.55^{b}	8.89 ± 0.08^{a}
NIP(Wx^{mp})	70.81 ± 0.89^{a}	10.57 ± 0.14^{c}	99.31 ± 3.95^{a}	66.15 ± 0.15^{b}	72.28 ± 0.13^{b}	79.05 ± 0.45^{b}	8.96 ± 0.03^{a}
2661(Wx^{b})	56.19 ± 0.85^{b}	17.14 ± 0.20^{a}	80.46 ± 1.80^{b}	63.81 ± 0.11^{a}	69.57 ± 0.34^{a}	74.70 ± 0.40^{a}	7.23 ± 0.96^{b}
2661(Wx^{mw})	66.29 ± 0.54^{a}	14.07 ± 0.01^{b}	95.99 ± 2.94^{a}	63.61 ± 0.49^{a}	69.41 ± 0.20^{a}	74.61 ± 0.60^{a}	8.53 ± 0.84^{a}

Data represent means ± standard deviation, n = 3. T_o, T_p, T_c and ΔH indicate onset temperature, peak temperature, conclusion temperature, and enthalpy of gelatinization, respectively. Means with different lowercase letters in each column for the same cultivar are significantly different by one-way ANOVA with multiple comparisons and student's *t*-test analysis ($p < 0.05$).

Table S5. Pasting properties of rice flours from near-isogenic lines (NILs) in the Nipponbare (NIP) and 2661 backgrounds

**Lines**	**Peak Visco (cP)**	**Trough Visco (cP)**	**Breakdown (cP)**	**Final Visco (cP)**	**Setback (cP)**	**Peak Time (min)**	**Pasting Temp (℃)**
NIP(Wx^{b})	2919.50±21.92^{a}	2022.50±16.26^{a}	897.00±5.66^{c}	3118.00±15.56^{a}	198.50±6.36^{a}	6.38±0.35^{a}	77.31±0.06^{a}
NIP(Wx^{mw})	2870.50±10.60^{b}	1442.00±28.28^{b}	1429.00±39.60^{a}	2026.00±33.94^{b}	-246.00±43.84^{b}	6.17±0.04^{a}	75.53±0.53^{b}
NIP(Wx^{mp})	2645.00±11.31^{c}	1293.00±18.38^{c}	1352.00±7.07^{b}	1899.00±9.89^{c}	-746.00±1.41^{c}	5.57±0.04^{b}	74.88±0.03^{b}
2661(Wx^{b})	2275.50±9.19^{a}	1758.00±7.07^{a}	517.50±16.26^{b}	2853.50±9.19^{a}	578.00±18.38^{a}	6.62±0.03^{a}	73.15±0.14^{a}
2661(Wx^{mw})	2067.50±13.43^{b}	1308.50±10.60^{b}	759.00±24.04^{a}	1852.50±6.36^{b}	-215.00±7.07^{b}	6.13±0.01^{b}	73.01±0.26a

Data represent means ± standard deviation, $n = 2$. For each column in the same cultivar background, values with different lowercase letters are significantly different by one-way ANOVA with multiple comparisons and student's t-test analysis ($p < 0.05$).

Table S6. Average genetic distances between groups carrying various *Wx* alleles

Groups	Numbers of accessions	Wx^{lv}	Wx^{a}	Wx^{in}	Wx^{b}	Wx^{mw}	Wx^{op}	Wx^{mp}	*wx*
Wx^{lv}	70	0							
Wx^{a}	89	0.00252							
Wx^{in}	93	0.00489	0.00489						
Wx^{b}	92	0.00486	0.00597	0.00065					
Wx^{mw}	4	0.00525	0.00636	0.00052	0.00044				
Wx^{op}	9	0.00168	0.00313	0.00553	0.00548	0.00590			
Wx^{mp}	3	0.00476	0.00572	0.00133	0.00084	0.00114	0.00532		
wx	10	0.00472	0.00586	0.00089	0.00039	0.00069	0.00526	0.00100	0

Population average distances were calculated from SNPs in 370 accessions (Table S8) using the bootstrap method.

Table S7. Moisture content of rice grains from different NILs after gradient drying as shown in Figure 3A

**Lines**	**Moisture content (%)**					
	**2 h**	**4 h**	**6 h**	**8 h**	**12 h**	**24 h**
NIP(*wx*)	18.09±0.67^{a}	14.97±0.78^{a}	13.90±0.69^{a}	12.27±0.59^{a}	11.46±0.20^{a}	10.10±0.22^{a}
NIP(Wx^{b})	17.15±0.41^{b}	14.18±0.42^{a}	13.93±0.51^{a}	12.18±0.35^{a}	11.76±0.11^{a}	10.55±0.19^{a}
NIP(Wx^{mw})	17.72±0.66^{b}	14.72±0.81^{a}	14.09±0.33^{a}	13.12±0.41^{a}	11.93±0.30^{a}	10.85±0.35^{a}
NIP(Wx^{mp})	17.12±0.18^{b}	15.27±0.54^{a}	13.73±0.75^{a}	12.98±0.77^{a}	11.65±0.23^{a}	9.99±0.15^{a}

The mature seeds were dried directly in a drying oven (40°C) for 2, 4, 6, 8, 12, and 24 h and then milled to white rice for measurement of moisture content. Data represent means ± standard deviations, n = 2. For each column, values with different lowercase letters are significantly different by one-way ANOVA with multiple comparisons ($p < 0.05$).

Table S8. List of rice accessions used in this study
